# Euthanasia

**Published:** 1906-02-24

**Authors:** 


### EUTHANASIA.

Euthanasia is a word common upon the tongues of men in all educated communities, for it cannot be denied that, to a reflective mind, no little of that apprehension which has been called the chief pain of death centres round a fear that the final dismissal may be preluded by a long and painful disablement. But although euthanasia, with the advantages of the proceedings commonly comprehended by the term, has long supplied food for argument as an abstract proposition, it has seldom commanded such a hardy advocate as Representative Hunt, of Cincinnati, who has introduced into the Ohio House of Representatives a Bill making it lawful "to kill a person suffering from intense pain and for whose recovery there is no hope."

The question involved is one the material and moral aspects of which are so diametrically opposed that the prospect of any reasonable agreement of society on the subject is not to be looked for, and this quite apart from the infinite obstacles which beset any attempt to reduce the theory of the matter to practice: but that any person should have been found ready to adventure himself upon such a task as the latter is a commentary upon a very noticeable change in public opinion as to the moral obligation which attaches to the endurance of pain. This is no place to argue a question trenching upon the confines of religion, but it is a certain matter that prayers for escape from sudden death find far less echo nowadays than they did half a century ago.

But whatever be the moral standpoint of individuals, to the medical profession at large the subject presents an abiding interest, for the circumstances of his daily life cannot fail to prompt in the mind of the practitioner a frequent query as to the correct ethical attitude towards sufferers in the last stages of irrecoverable disease. What, for instance, is the correct course of conduct for the physician who is called to attend a patient who—to put an extreme case—after suffering the pangs of angina pectoris for a twelvemonth or more, has passed into a condition of uræmic or diabetic coma? The practitioner is aware, let us assume, that his patient is weary of living under the unfortunate conditions in which he finds himself: he is aware, on the other hand, that by the use of active measures, such as the infusion of saline solution, he may be able to retard, perhaps to banish for a time, the imminent risk of dissolution. Is he to employ his knowledge or to hold his hand? Cognate examples will readily present themselves to all who have had much experience of death-beds, and the answer in any given case is, it seems to us, a matter for the individual conscience.

We have purposely stated a case of extreme difficulty in order to demonstrate the potential subtleties of the problem, but the general attitude of the profession must undoubtedly remain, as it has always been, devoted to the preserving of life for as long a time as possible. To admit the general right of the physician to dispense life and death is to charge him with moral responsibilities which few physicians would consent to assume. It would, moreover, open the door to sentimental faddists in certain matters closely affecting the welfare of the State. For example. It is not infrequent to hear from kind-hearted people the opinion that high infantile mortality is an unmixed blessing, as yearly relieving several thousands of souls from the distresses of what would under the circumstances probably have been a life of poverty. Such an attitude of morbid pessimism may be good "Hamlet," but it has no place in practical sociology.

The truth is that there are few unpleasant situations in the lives of men which are in realisation as bad as imagination paints them, and plenty of people who are afflicted with incurable disorders none the less derive a vast degree of pleasure from living, and contribute immensely to the service of the State. If the medical profession means to avoid a position of sterilising hopelessness its duty in the face of incurable diseases is perfectly clear in the main. Such diseases are almost without exception capable of mitigation, and, all other therapeutic means apart, respond in a surprising degree to the promptings of hope. There is no weapon in the armament of medicine which is more essential than optimism, nor one which in the face of apparently overwhelming odds is capable of giving so good an account of itself. We cannot all be optimists by conviction, but a determined soul will assume the virtue if he has it not, for in it is the foundation of success. Euthanasia, in the popular meaning of the word, may safely be left to philosophers and the legislators of Ohio who allowed the Euthanasia Bill to be introduced by a vote of 79 to 23. Philosophy is very well, but too much philosophy breeds a detachment which is inimical to practical progress whether in the art of government or of medicine, and if medicine be not practical it has lost its justification for existence.

